# Clarifying the misinterpretation of case-fatality rate as mortality in thyroid cancer studies

**DOI:** 10.1097/JS9.0000000000002133

**Published:** 2025-03-14

**Authors:** Kyu-Won Jung, Eun Hye Park, Mee Joo Kang

**Affiliations:** a Korea Central Cancer Registry, National Cancer Center, Goyang, Korea; b Division of Cancer Registration and Surveillance, National Cancer Control Institute, National Cancer Center, Goyang, Korea

*Dear editor*,

We would like to address several important points regarding the article “*Thyroid Cancer-Specific Mortality During 2005–2018 in Korea: Aftermath of the Overdiagnosis Issue: A Nationwide Population-Based Cohort Study*”^[^1^]^ recently published in *the International Journal of Surgery*. While this study provides valuable insights into thyroid cancer incidence and mortality trends in Korea, we are concerned that the use and interpretation of thyroid cancer-specific mortality may cause confusion and lead to misinterpretation of the results.

First, the authors use the term “thyroid cancer-specific mortality,” but the analysis is based on deaths among thyroid cancer patients rather than the general population. This would be more appropriately termed “case fatality rate” (CFR). As outlined in the epidemiological literature^[^2,3^]^, mortality rate refers to the number of deaths in the general population during a specified interval of time, while the CFR reflects the proportion of deaths among individuals diagnosed with the disease. This distinction is crucial because mortality assesses the burden of disease on a population, while CFR focuses on outcomes within an already diagnosed group of patients. and measured the severity of the disease. Furthermore, while the CFR is an appropriate measure for acute diseases owing to its rapid progression and higher immediate fatality risk, its application to chronic diseases such as cancer can be misleading. In the case of thyroid cancer, the risk of death from cancer itself is low, and the probability of death from other causes increases over time.

Second, this study calculated CFR as a rate using person-years, which assumes a constant risk over time. However, for cancer patients, the risk of death is highest within the first year after diagnosis and decreases with longer follow-up. After approximately 5 years, the risk of death is generally minimal. Additionally, the shorter follow-up periods for patients diagnosed in later years (e.g., 2018) compared to those in earlier years (e.g., 2013) may have inflated the CFR, as the highest risk occurred shortly after diagnosis.

Third, the authors noted an increase in thyroid cancer-specific CFR after 2013, despite the overall decrease in incidence. However, we interpret that this increase in CFR could be attributed to changes in the case mix following a reduction in the overdiagnosis of low-risk thyroid cancer cases. As screening for low-risk populations decreases, the proportion of high-risk patients likely increases. Consequently, the observed increase in CFR may reflect a higher proportion of advanced cases, rather than a genuine increase in CFR. This case-mix shift is supported by data from Oh *et al*^[^4^],^ which showed a significant reduction in thyroid cancer incidence due to changes in screening practices. Similarly, Cancer Statistics in Korea: Incidence, Mortality, Survival, and Prevalence in 2021 reported that the age-standardized mortality rate for thyroid cancer in the general population decreased from 1.06 per 100 000 in 2012 to 0.80 per 100 000 in 2018^[^5^]^. This suggests a decline in overall mortality despite the increased CFR observed in the patients diagnosed in this study. As shown in Table 1, the proportion of patients diagnosed with localized versus regional/metastatic thyroid cancer shifted significantly after 2013, with an increase in the relative proportion of advanced-stage cases. This shift in the patient population further supports the argument that the increase in CFR may be due to changes in the case mix rather than a true rise in thyroid cancer-specific mortality. An increase in the proportion of high-risk patients within the thyroid cancer cohort could explain the increase in all-cause CFR and CFR observed among patients who underwent total thyroidectomy in this study.

**Table 1:** Trends in incidence and mortality of thyroid cancer in Korea by SEER Summary Stage, 2005–2018.

Year	New cases (*N*)	Incidence			Age-standardized incidence rate per 100 000	Mortality	
		SEER Summary Stage			
		Total^a^(*N*)	Localized (%)	Regional/Metastatic (%)		Deaths (*N*)	Age-standardized mortality rate per 100 000
2005	12 833	10 307	50.4	49.6	31.2	330	1.28
2006	16 200	13 736	50.3	49.7	38.2	332	1.24
2007	21 331	18 505	49.4	50.6	49.4	381	1.38
2008	27 373	24 459	48	52	62.0	347	1.22
2009	32 586	28 773	47.7	52.3	72.0	353	1.15
2010	36 873	34 117	45.5	54.5	79.8	356	1.14
2011	41 396	38 598	46.8	53.2	88.2	390	1.18
2012	44 798	42 291	46.5	53.5	94.3	376	1.06
2013	43 124	40 285	44.5	55.5	88.8	394	1.08
2014	31 349	28 931	43.4	56.6	63.7	346	0.89
2015	25,576	23 616	42.3	57.7	51.4	341	0.85
2016	26 679	24 719	42	58	53.1	346	0.82
2017	26 843	25 154	42.7	57.3	53.0	369	0.83
2018	29 070	27 186	47.2	52.8	57.0	372	0.80

^a^ Patients with an unknown SEER Summary Stage were excluded from the study

In conclusion, we propose that the findings presented in this study should be re-evaluated in light of the distinction between thyroid cancer-specific mortality and CFR. The observed increase in CFR is likely due to the changing case mix of patients and unequal follow-up periods, rather than a true increase in mortality rate. Accurate terminology and consideration of case-mix shifts are essential to understand the real impact of overdiagnosis and treatment practices on thyroid cancer outcomes. We believe that these clarifications are crucial for accurately interpreting the findings of this study and their implications for clinical practice.

## Data Availability

No data was generated during the writing of this study.

## Research Registration Unique Identifying Number (UIN)

1. Name of the registry: not applicable.
2. Unique identifying number or registration ID: not applicable.
3. Hyperlink to your specific registration (must be publicly accessible and will be checked): not applicable.
